# The Challenges and Strategies of Antisense Oligonucleotide Drug Delivery

**DOI:** 10.3390/biomedicines9040433

**Published:** 2021-04-16

**Authors:** Maria Gagliardi, Ana Tari Ashizawa

**Affiliations:** Bio-Path Holdings, Inc., Bellaire, TX 77401, USA; mgagliardi@biopathholdings.com

**Keywords:** antisense oligonucleotide, liposomes, drug delivery, Grb2

## Abstract

Antisense oligonucleotides (ASOs) are used to selectively inhibit the translation of disease-associated genes via Ribonuclease H (RNaseH)-mediated cleavage or steric hindrance. They are being developed as a novel and promising class of drugs targeting a wide range of diseases. Despite the great potential and numerous ASO drugs in preclinical research and clinical trials, there are many limitations to this technology. In this review we will focus on the challenges of ASO delivery and the strategies adopted to improve their stability in the bloodstream, delivery to target sites, and cellular uptake. Focusing on liposomal delivery, we will specifically describe liposome-incorporated growth factor receptor-bound protein-2 (Grb2) antisense oligodeoxynucleotide BP1001. BP1001 is unique because it is uncharged and is essentially non-toxic, as demonstrated in preclinical and clinical studies. Additionally, its enhanced biodistribution makes it an attractive therapeutic modality for hematologic malignancies as well as solid tumors. A detailed understanding of the obstacles that ASOs face prior to reaching their targets and continued advances in methods to overcome them will allow us to harness ASOs’ full potential in precision medicine.

## 1. Introduction

The correlation between differential gene expression and disease has revolutionized molecular and clinical research, allowing the identification of disease biomarkers and therapeutic targets [1]. Oligonucleotide therapeutics have since developed to specifically silence, restore, or modify the expression of disease-causing or disease-associated genes in cancer and genetic disorders [2,3]. These therapeutics include antisense oligonucleotides (ASOs), small interfering RNA (siRNA) and microRNA that interfere with coding and noncoding RNA; aptamers and decoys, which rely on their secondary structure to bind to and compromise protein function; and CRISPR/Cas9, a promising gene editing technology that directly targets genomic DNA [4,5]. Oligonucleotide technology has broadened the spectrum of possible therapeutic targets. By controlling protein expression via RNA interference, previously “undruggable” proteins that were unaffected by conventional small molecules can now be affected.

In this review we will specifically focus on ASOs, as they are the most clinically developed, with several drugs already approved by the U.S. Food and Drug Administration (FDA) and in clinical trials. siRNA and microRNA are also inundating clinical pipelines and are proving to be efficacious; however, they come with specific challenges that will become more evident as we describe ASOs. ASOs are single-stranded chemically modified nucleic acid polymers, typically in the range of 18–25 bases in length. Once bound to the target RNA sequence via Watson–Crick base pairing, the RNA can be degraded, hindered, or manipulated by alternative splicing [6]. This makes ASOs more versatile than siRNA or microRNA because ASOs, in addition to reducing protein expression, can also enhance target translation. Another advantage of these therapies is that due to their straightforward design and production, the time between their conceptualization and clinical use is reduced. This is evidenced by their escalating presence in drug pipelines [7]. siRNA is double stranded, a feature that while making it more potent than ASOs also makes its design and manufacture more complex and costly, and more prone to inducing toxicities due to activation of innate immune response via Toll-like receptors and off-target effects [8,9,10]. Finally, the ease and accuracy with which ASO sequences can be customized allows virtually any mutated gene to be targeted, creating great progress in precision and personalized medicine [11]. Milasen, for example, is a 22-base ASO that was designed to treat a single six-year-old patient with Batten disease, a rare and fatal neurodegenerative disease. The MGSD8 gene of this patient was found to harbor a novel and pathogenic mutation. Just one year after the identification of this mutation Milasen was created and treatment began, resulting in the reduction of symptoms and overall improved quality of life [12]. The use of microRNA in such a setting would not be as useful as ASO. microRNA is able to bind to target RNA via incomplete base pairing, thus allowing it to simultaneously silence multiple targets [13]. Although this may be advantageous if the downregulation of a specific signaling pathway rather than a specific protein is desired, it could produce off-target effects and toxicities [14,15].

Albeit a promising technology, the delivery of ASO therapies to specific tissues and cellular uptake poses a great challenge and limitation. Liposomal delivery is one of several strategies used to overcome such challenges. Liposomes improve ASO stability in biological fluids, promote drug distribution, enhance cellular uptake, and escape the endocytic pathway [16,17]. BP1001, a liposome-incorporated antisense oligodeoxynucleotide targeted against growth factor receptor-bound protein-2 (Grb2), is a unique, non-toxic anti-cancer drug currently in a phase 2 clinical trial. Grb2 is an ideal drug target as it is a ubiquitously expressed adaptor protein that links activated growth factor receptors to various downstream oncogenic signaling pathways involved in cell growth, proliferation, and metabolism [18,19].

This review will describe the challenges of ASO drug delivery and the multiple strategies being explored to address them. A detailed overview of BP1001′s pre-clinical and clinical data will demonstrate the advances made in liposomal drug delivery and highlight its promising potential in cancer therapeutics.

## 2. ASO Mechanisms of Action and FDA-Approved Drugs

### 2.1. Mechanisms of Action

**Figure 1:**
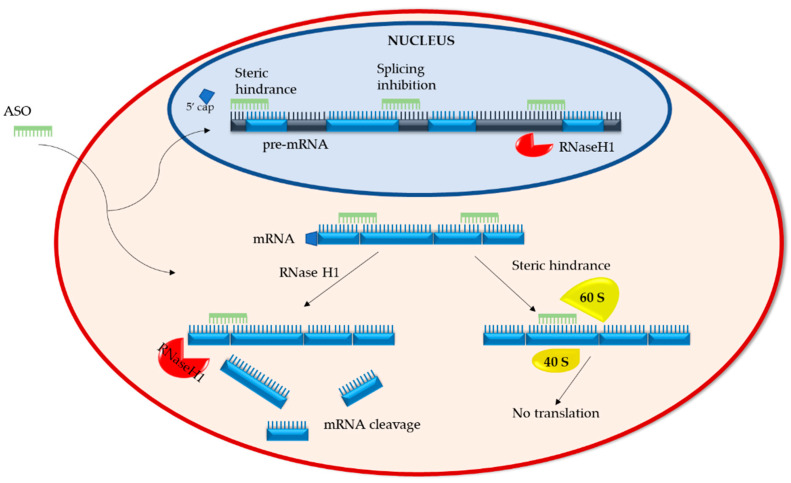
Antisense oligonucleotide mechanisms of action. ASOs interfere with pre/mRNA in two ways: (i) by promoting their cleavage via RNaseH1 and (ii) by blocking processing events such as 5′capping of the pre-RNA, exon splicing, and the interaction of mRNA with ribosomal subunits (40S and 60S) of the translation initiation complex.

ASOs have two distinct mechanisms of action. The first entails the degradation of the RNA and the second involves the inhibition or modulation of RNA via steric hindrance (Figure 1).

#### 2.1.1. RNA Degradation

Upon ASO binding to target RNA, RNA/ASO duplexes are formed. These duplexes recruit ribonuclease H1 (RNaseH1), an endonuclease that cleaves the phosphodiester bonds of RNA. This ASO mechanism is active both in the cytoplasm, where mRNA is targeted, and the nucleus, where non-coding and pre-mRNA are targeted [20]. Post RNaseH1 cleavage, RNA fragments are degraded by XRN exonucleases and the exosome complex, which are standard RNA degradation pathways in both the cytoplasm and nucleus [20,21,22]. Studies have shown that overexpression of RNaseH1 in Hela and A549 cells increases the potency of ASOs, whereas reduction of RNaseH1 levels reduces ASO efficacy, making RNaseH1 a rate limiting factor in ASO activity [23,24]. Though generally considered a potent and reliable mechanism with prolonged duration, a recent paper by Liang et al. describes the development of ASO tolerance. They found that as the recruited RNase H1 cleaves target mRNA in ribosomes, the levels of pre-RNA of the same gene increase due to enhanced transcription, thereby abrogating ASO gene silencing. This process of tolerance is RNaseH1 dependent and target-sequence specific [25]. 

#### 2.1.2. RNA Steric Hindrance

Sugar modifications (such as 2′-O-methyl (2′-OMe) or 2′-O-methoxyethyl (2′-O-MOE)) added to phosphorothioate-modified ASOs render the RNA/ASO duplexes resistant to endonuclease cleavage (please refer to Section 3.1 for “Chemical modifications of ASOs”) [26]. The binding of such duplexes within the 5′-untranslated region (5′UTR) of mRNA sterically blocks mRNA translation by interfering with the assembly of the initiation complex (40S and 60S ribosomal subunits) at the AUG start codon [27]. Steric hindrance can also regulate the processing of pre-mRNA in the nucleus by preventing 5′ capping or directing splice site selection [28]. Depending on the binding site, splice selecting ASOs can prevent splicing factors from accessing the transcription site or inducing the expression of abnormal, non-functional proteins. 

In addition to reducing target gene expression, steric hindrance ASOs can also enhance the expression of specific therapeutically advantageous proteins [29]. Indeed, ASOs designed to hybridize and block upstream open reading frames (uORF) in mRNA enhance the interaction of the pre-initiation complex with the primary ORF (pORF), thereby increasing translation efficiency and protein expression [29,30]. Alternative splicing can also induce the expression of therapeutic splice variants. For example, splice shifting of Bcl-x pre-mRNA to increase the Bcl-x_S_ levels (pro-apoptotic) at the expense of Bcl-x_L_ (anti-apoptotic) was shown to bolster the apoptosis of hepatic stellate cells, suggesting a therapeutic approach for liver fibrosis, and increased the sensitivity of MCF-7 (breast cancer) and PC3 (prostate cancer) cells to chemotherapeutic agents [31,32].

Thus, the chemistry and target sequence of ASOs determines their mechanism of action and the fate of the targeted RNA.

### 2.2. FDA-Approved ASO Drugs

To date there are eight FDA-approved ASO drugs (excluding Milasen, which is patient specific). Three are RNaseH1 dependent and five are splice-altering ASOs. 

#### 2.2.1. RNaseH1-Dependent ASOs

Formivirsen (ISIS 2922) was the first ASO drug approved by the FDA in 1998 as a therapy for cytomegalovirus (CMV) retinitis in immunocompromised AIDS patients [33,34]. Classified as first-generation ASO, the natural phosphodiester linkage in the DNA backbone is replaced with a phosphorothioate linkage, making the oligonucleotides more resistant to nuclease degradation. Intravitreal administration of Formivirsen delays CMV replication, showing efficacy in clinical trials both alone and in combination with other antiretroviral drugs [35]. In 2002 and 2006 Formivirsen was withdrawn from the EU and US markets, respectively. The development of combination antiviral therapeutics replaced the need of Formivirsen due to a reduction in additional patient infection [36].

Mipomersen is a second-generation ASO with 2′-O-MOE-based nucleotides and a chimeric GAPmer design. Targeting apolipoprotein B mRNA, it is used for the treatment of homozygous familial hypercholesterolemia, a rare genetic disorder characterized by high levels of low density lipoprotein (LDL) cholesterol [37]. In a randomized, double-blind, placebo-controlled phase 3 clinical trial, patients treated with a once-weekly subcutaneous Mipomersen injection displayed a significant reduction of LDL cholesterol compared to the placebo group [38]. Despite these promising results, Mipomersen was not approved by the European Medicines Agency due to concerns of potential liver damage. The FDA approved it in 2013; however, its use is reduced due to safety concerns and the availability of other treatment options [39].

Inotersen, like Mipomersen, is a second-generation ASO with 2′-O-MOE-modified nucleotides and GAPmer structure. It was approved in 2018 for the treatment of transthyretin amyloidosis [40,41]. Transthyretin is plasma protein produced in the liver that forms a tetrameric complex for the transport of thyroid hormone thyroxin and the retinol binding protein complex throughout the body. Single-nucleotide polymorphisms in both the coding and non-coding regions of the transthyretin gene cause misfolding of the protein, resulting in amyloid deposits in different organs [42,43]. A randomized, double-blind, placebo-controlled phase 3 clinical trial revealed that patients treated with a weekly subcutaneous injection of Inotersen had lower levels of circulating transthyretin, slower disease progression, and improved quality of life [44].

#### 2.2.2. Splice-Altering ASOs

Nusinersen is a 2′-O-MOE-modified ASO approved by the FDA in 2016 for the treatment of spinal muscular atrophy (SMA), a neuromuscular disorder characterized by weakness in skeletal, bulbar, and respiratory muscles [45]. SMA is caused by the loss of survival of motor neuron 1 (SMN1) expression. Although this loss of expression could be rescued by the expression of paralogous gene SMN2, the frequent skipping of exon 7 in this gene during pre-mRNA splicing produces short, rapidly degraded proteins [46,47]. Nusinersen binds and modifies the splicing of SMN2 pre-mRNA to include exon 7, thus restoring full-length SMN expression [47,48]. Children with SMA who received Nusinersen treatment showed increased levels of SMN2 expression and greater motor function [49].

Eteplirsen received accelerated approval by the FDA in 2016 for the treatment of Duchenne muscular dystrophy (DMD) [50]. It is a third-generation phosphorodiamidate morpholino oligomer (PMO) ASO. The bases in this oligonucleotide are attached to a neutral morpholino ring and nucleotides are linked via phosphoramidate bonds [51]. These modifications attribute PMO-ASO with increased nuclease resistance, efficacy, and cellular uptake [51]. DMD is a rapidly progressing neuromuscular disorder. Mutations in the *DMD* gene that disrupt its reading frame introduce premature stop codons, halting gene expression. Eteplirsen repairs the reading frame by binding to exon 51 in the pre-mRNA, which is then “skipped” in translation, producing a shortened but functional dystrophin [52,53].

Golodirsen is another PMO-ASO, approved in 2019 for the treatment of DMD [54]. Mechanistically similar to Eteplirsen, Golodirsen adjusts the splicing pattern of mutated dystrophin pre-mRNA by binding to exon 53. The absence of exon 53 in the mature mRNA restores dystrophin expression [55]. A randomized, double-blind, placebo-controlled phase 1/2 clinical trial reported Golodirsen’s ability to safely increase dystrophin expression by ~16 fold. Additional studies will assess the impact that this increase has on disease progression [55].

Viltolarsen is another approved (2020) PMO-ASO that binds to exon 53 of dystrophin pre-mRNA for the treatment of DMD patients with genetic mutations that are amenable to exon 53 skipping [56,57]. Clinical trials in different countries provided consistent results. Specifically, DMD patients treated with either high or low doses of Viltolarsen all showed de novo dystrophin expression and exon 53 skipping [58,59]. Disease progression was slowed with higher dose treatments and no serious adverse events were observed [58,59].

Casimersen is the most recent PMO-ASO that received accelerated approval for the treatment of DMD in 2021 [60]. It differs from its predecessors by binding to and promoting the skipping of exon 45 [61]. In a double-blind study patients treated with Casimersen at 30 mg/kg for 48 weeks had increased dystrophin protein levels and did not experience any severe adverse effects [62]. Casimersen’s ability to improve motor function has yet to be established.

The approval of eight ASO therapies after decades of design and clinical development proves that the great potential of this technology is accompanied by complex challenges. Drug delivery to target tissues and cells is a prominent obstacle in treatments. Many strategies are currently being explored to improve the stability, distribution, and cellular uptake of ASOs.

## 3. Strategies to Optimize ASO Drug Delivery

One of the greatest challenges in ASO therapies is the delivery of the drug to the site of action. Upon injection, ASO drugs must travel though the blood stream, pass through biological barriers, and be internalized by cells. Once internalized, ASOs must escape from lysosomal degradation and avoid entrapment in secretory vesicles whose cargo is exported from cells [63,64]. Different strategies have and continue to be developed to improve ASO stability, targeting, and trafficking [65]. These include chemical modifications, bioconjugation to different moieties, and the use of delivery vehicles.

### 3.1. Chemical Modifications of ASOs 

All the FDA-approved ASO drugs described above are bare, untagged oligonucleotides. With no aid from delivery vehicles or escort bioconjugates, they rely on chemical modifications to remain stable and functional. 

The first chemical modification, establishing the first of three generations of ASOs, is of the inter-nucleotide phosphate group [7,66]. A non-bridging oxygen atom of this group is replaced by a sulfur atom, creating a phosphorothioate (PS) backbone [66]. PS-ASOs have a longer half-life, as they are not only nuclease resistant but also interact with plasma proteins such as albumin, which reduces their renal clearance [67]. Intracellularly, PS-ASOs also bind to a variety of proteins throughout the endocytic pathway, which can either enhance or impede their activity [68,69].

Second-generation ASOs, in addition to a PS backbone, have ribose sugar modifications such as 2′-OMe, 2′-MOE, or 2′-fluoro, which increase their binding affinity and nuclease resistance [70,71,72]. As mentioned above, these sugar modifications do not mediate RNaseH1 degradation [26]. GAPmer-designed ASOs, in which a central portion of PS nucleotides is flanked on either side by sugar-modified nucleotides, couples the degradation capacity of RNaseH1-recruiting nucleotides with the enhanced binding affinity and endosome resistance of sugar-modified ones [73]. Recent work has furthered the use of sugar-modified nucleotides by showing that the incorporation of fluorinated nucleotides at selected positions within the gap region itself can direct the cleavage of specific gene alleles with single-nucleotide polymorphisms [74,75]. 

Third-generation ASOs that also follow a GAPmer design surpass previous generations in terms of nuclease resistance, binding affinity, cell penetration, efficacy, and reduced off-target effects [73]. The chemical modifications in this group include: 

Nucleobase modification. An example of nucleobase modifications is the methylation of 5′ cytosines. This increases the hydrophobicity of ASOs, thereby enhancing their affinity to target RNA [76,77]. This modification has been implemented for the improvement of a Superoxide Dismutase 1-targeting ASO currently in clinical trial for the treatment of amyotrophic lateral sclerosis [78,79]. 

Bridged nucleic acids. The conformationally restrictive bridging of the 2′ and 4′ carbons of the ribose sugar characterize locked nucleic acids (LNA) and 2′-O-ethyl (constrained ethyl) (cEt) bicyclic nucleic acids (BNA). Unable to recruit RNaseH1, cEt-BNAs are found in the flanking regions of a GAPmer to enhance target binding [80]. Although LNAs are also placed in GAPmer flanking regions to enhance binding, it has been shown that mixed LNA/DNA oligonucleotides retain RNaseH1 activating ability and are significantly less affected by exonucleases compared to unmodified oligonucleotides [64,81]. They have also shown to be efficient at optimizing exon skipping during pre-mRNA splicing [82]. However, serum transaminase elevation, indicative of hepatotoxicity, has been associated with LNA-modified ASOs [83,84].

Alternative backbones. As described in Section 2.2.2, PMO have a six-membered morpholino ring stringed together by phosphoramidate bonds and operate via steric hindrance in RNA processing and translation. The fact that the four most recent FDA-approved ASO drugs are PMO based is testimony to their elevated efficacy, enzyme resistance, and low toxicity [55,57,62]. Ethoxy-modified oligonucleotides, which are currently in clinical trials, have an ethyl group added to a non-bridging oxygen atom in the phosphate backbone. Contrary to PS backbones, P-ethoxy backbones are lipophilic and do not interact with charged cellular proteins, thereby reducing potential toxicities [85]. Peptide nucleic acids, another synthetic DNA mimetic, have a pseudo peptide polymer backbone with high binding affinity and stability [86]. A commonality of the above alternative backbones is that they all preclude RNase activity and are uncharged; although this promotes affinity and binding it is also a limitation due to reduced cellular uptake, requiring the development and use of delivery systems [86].

### 3.2. Bioconjugation and Targeting

With the knowledge of chemical modifications required to protect ASOs from exonucleases and prolonging their stability, the next challenge becomes ASO passage across biological barriers, such as the vascular endothelial barrier, cell membranes, and intracellular compartments [65]. The liver, eyes, and central nervous system are considered the easiest target sites thus far and have been the focus of most therapeutics (e.g., Inotersen, Formivirsen, Nusinersen) [87]. The gaps along the endothelial linings of the liver tissue and the direct injection of ASO into the eye and spinal cord favors the distribution and accumulation of ASO drugs at these sites [87,88]. Strategies to improve and expand ASO delivery potential involve the conjugation of ASOs to different moieties that can direct the drug to specific tissues and enhance internalization.

Peptide conjugates are short cationic or amphipathic peptides able to cross cell membranes by either direct penetration or by interacting with specific cell receptors that are endocytosed [89,90]. These peptides can be designed to become functionally active once internalized, causing endosome disruption and drug release into the cytoplasm [89]. Pip6a is an example of a cell-penetrating peptide (CPP). Composed of a hydrophobic core flanked by arginine-rich domains, it displays serum stability and efficient crossing of plasma and endosomal membranes. When bound to neutral PMO targeting dystrophin pre-mRNA, the appropriate exon skipping occurs to increase dystrophin expression for DMD therapy [91,92]. 

Antibody conjugates, already used for the delivery of cytotoxic drugs in different cancer therapies, are a promising strategy for cell targeting and enhanced internalization of ASO drugs. Still in early stages of development, pre-clinical studies show that the expression of a key target protein in glioblastoma cancer stem cells, DRR/FAM107A, can be reduced when treated with ASOs conjugated to an anti-CD44 monoclonal antibody (mAb) as a result of increased cellular uptake [93]. Likewise, anti-CD22mAb conjugated to MXD3-ASO reduced the expression of MXD3, thereby promoting the apoptosis of precursor B-cell acute lymphoblastic leukemia cells in a dose-dependent manner (both in vivo and in vitro) [94]. 

Aptamers are oligonucleotide sequences ranging from 25 to 100 bases that adopt diverse 3D structures to bind to target cell surface proteins. Mimicking antibody function in terms of protein binding, they have the advantage of being low cost, easily designed, and conformationally flexible, and have low immunogenicity [95]. Recently, non-specific cell internalization aptamers were generated with the addition of artificial 5-((3-indolyl)propionamide-N-allyl)-20-deoxyuridine (U^trp^) in place of deoxythymidine. Although their increased hydrophobicity enhanced internalization, U^trp^aptamer-ASO did not increase ASO activity due to endosomal retention. This suggests that the membrane proteins that aptamers interact with are of great importance as they determine their intracellular trafficking and activity [95]. 

Acetylgalactosamine (GalNAc) binds to the highly expressed asialoglycoprotein receptor 1 (ASGR1) in hepatocytes. The role of ASGR1 is to clear glycoproteins from the blood through clathrin-mediated endocytosis. Although the liver is an easily accessible ASO target site, the conjugation of ASO drugs to GalNAc enhances their potency by providing a reliable entry route. Once internalized, the GalNAc-ASO is metabolized at different sites to release the ASO in the cytosol. Early studies in a mouse model showed that a GalNAc-ASO targeting human transthyretin was 10 times more potent and was active for a longer duration than unconjugated ASO [96]. Phase 2 clinical trials of GalNAc-ASO-targeting apolipoprotein A in patients at risk of cardiovascular disease also showed a significant decrease in protein expression [97]. These results and the FDA approval of Givosiran, a GalNAc-conjugated siRNA for the treatment of acute hepatic porphyria, make GalNAc a popular strategy for ASO delivery to hepatocytes.

### 3.3. Delivery Vehicles

As the field of nanotechnology rapidly advances, many different nano-drug delivery vehicles are being explored, from DNA nanostructures to exosome-like nano-carriers to spherical nucleic acids, all described in recent reviews [98,99,100]. In this review, we will focus on liposomal drug delivery.

**Figure 2:**
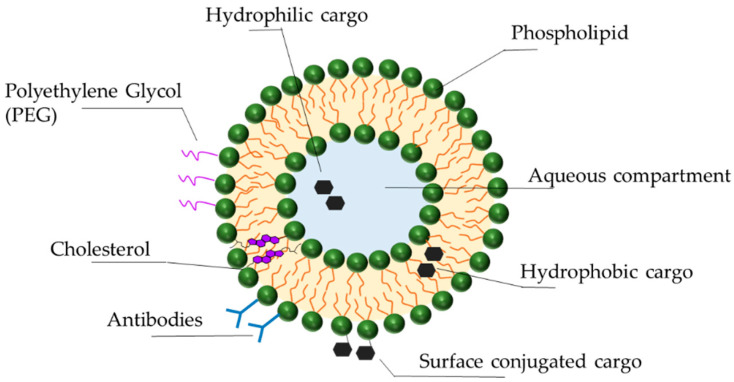
Liposomal delivery vehicles. Liposomes are spherical drug delivery vehicles made of a phospholipid bilayer with an aqueous core. Its hydrophobic and hydrophilic nature accommodates hydrophobic cargo in between the lipid layers and hydrophilic cargo in the aqueous compartment or conjugated to the external lipid polar headgroups. The polyethylene glycol (PEG) coating of liposomes prolongs their circulation by reducing uptake by macrophages. Surface labeling with antibodies promotes liposomal targeting to specific cells and cellular uptake. Cholesterol in the hydrophobic domain of the bilayers affects liposomal rigidity and permeability.

Liposomes are lipid vesicles composed of a natural or synthetic lipid bilayer surrounding an internal aqueous compartment (Figure 2). First identified in the 1960s, they have since become the primary focus of nanoparticle therapeutics, offering significant advantages regarding stability, selective delivery, low toxicity, and biocompatibility. Diverse liposome formulations are currently in clinical trials. The combination and unique features of their components (lipids, synthetic amphiphiles, sterols) result in liposomes of different charges, sizes, and efficacies [101,102]. The following describes some of the latest advances in liposomal technology for the optimization of drug delivery.

While protecting ASOs from nucleases, liposomes themselves are exposed to the destabilizing effects of serum proteins, such as opsonins and lipoproteins (HDL, LDL). Opsonins in the blood detect and bind to liposomes, priming them for phagocytosis [103,104,105]. Lipoproteins structurally destabilize liposomes by depleting them of lipids, resulting in release of the cargo [106]. To reduce these protein interactions and immunogenicity, polymers such as poly(ethylene glycol) (PEG) are covalently attached to the surface of the liposomes (PEGylation), creating a stealth effect [107,108]. Initially thought to reduce non-specific cellular uptake by directly creating steric hindrance, recent work shows that only PEGylated liposomes that have been exposed to serum proteins have reduced macrophage uptake. Those that have not been exposed to serum proteins remain macrophage targets [109,110]. It is therefore the biomolecules in the serum that interact with PEGylated liposomes that protect them from immune detection. Although PEGylation is a commonly used and promising technology, it does have drawbacks. PEG is non-biodegradable; its accumulation in the body can lead to adverse reactions and hypersensitivity [110,111]. In addition, the formation of PEG antibodies (anti-PEG IgM) after the first administration of PEGylated liposomal drugs can compromise the efficacy of subsequent administrations [105]. In the presence of anti-PEG IgM, PEGylated liposomes are recognized by the mononuclear phagocytic system and undergo accelerated blood clearance [105,108,112]. Alternatives to PEGylation currently being explored include poly(glycerols), poly(oxazolines), and poly(zwitterions) [110,113].

Cholesterol is an important component of the liposomal bilayer, contributing to its function in a variety of ways. By inducing dense packing of the phospholipids within the bilayer it protects liposomes from HDL activity, thus enhancing liposomal stability and circulation [114,115]. Lipid density also makes the liposomes less permeable to small molecules, ions, and the drug cargo itself, ensuring protection and drug retention [116]. Finally, cholesterol reduces the binding of Na+ ions to the liposome, which in turn reduces liposomal interaction with serum opsonins, once again promoting immune evasion and increasing circulation time [107,115,116]. 

Just as ASOs are conjugated to targeting moieties such as peptides, aptamers, or antibodies, these moieties can also be attached to liposomes. These additions combine the benefits of cell targeting with liposomal drug stability. In a proof-of-concept study an innovative immunoliposome (iLi) carrying a Translationally Controlled Tumor Protein-targeting ASO and functionalized with the addition of the Her2 antibody was created for the treatment of prostate cancer [117]. Results show that the ASO-iLi had a greater antiproliferative effect on PC-3 cell spheroids than ASO liposomes [117]. Interestingly, this effect required long exposure times, indicating that increased steric hindrance due to antibody conjugation could delay ASO release. This higher efficacy and slow release could make it an ideal candidate for treatments with prolonged exposure [117]. 

Novel stimuli-sensitive liposomes are designed to release their cargo in response to specific pathophysiological stimuli. They rely on the use of synthetic lipids that, once triggered, undergo conformational changes to disrupt the liposomal membrane. These stimuli can be endogenous, such as pH and redox potential changes, or they can come from an external source such as temperature, ultrasound, and magnetic fields [118,119]. For example, pH-sensitive liposomes, once endocytosed, are triggered by the decrease in endosomal pH to fuse with the endosomal membrane for the release of their cargo into the cytoplasm [120]. Additionally, the pH-sensitive element of the liposome can be used to improve cellular uptake. A pH-sensitive ‘activatable cell-penetrating peptide’ (ACPP) was developed, consisting of an inhibitory peptide that shields the CPP under normal physiological pH [121]. Once located at disease sites characterized by lower pH, such as tumors, the ACPP undergoes acid-catalyzed cleavage. The detachment of the inhibitory peptide exposes the CPP, thus promoting the internalization of the liposome [119,121].

Due to their improved stability, biocompatibility, extended circulation time, controlled release, and efficacy, there are many clinical trials involving liposomal drugs [122]. One of the main disease targets of these drugs is cancer. Since the initial discovery of oncogenes, our understanding of cancer genomics has increased exponentially. The Cancer Gene Atlas now allows us to identify appropriate genetic targets and has propelled the development of oligonucleotide therapeutics [123,124,125].

## 4. BP1001: Liposomal P-Ethoxy-Grb2 ASO

**Figure 3:**
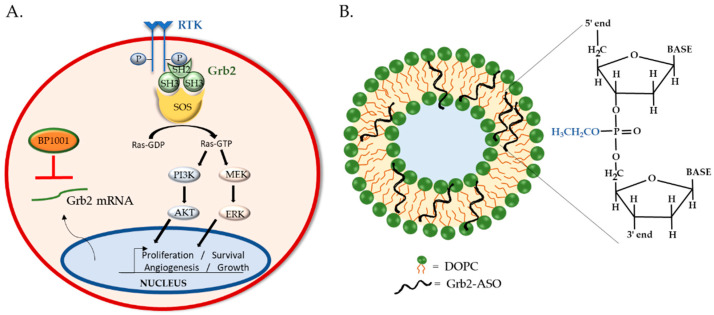
Grb2 signaling and BP1001 interference. (**A**) Grb2 is an adaptor protein that binds to phosphorylated receptor tyrosine kinases and mediates the activation of Ras via the recruitment of guanine exchange factor SOS. Ras activation initiates MAPK/ERK and PI3K/AKT signaling. (**B**) BP1001 is a liposomal-incorporated Grb2-ASO with a P-ethoxy modified backbone. It precludes Grb2 protein expression by blocking mRNA translation.

Grb2 is a ubiquitously expressed, non-enzymatic adaptor protein that mediates the interaction of receptor tyrosine kinases (RTKs) with downstream proteins involved in cell proliferation, differentiation, motility, angiogenesis, and apoptosis [126,127]. Grb2 is instrumental in the regulation of oncogenic processes such as tumor progression, metastasis, and drug resistance [128]. Canonically, Grb2 binds to auto-phosphorylated tyrosine resides of activated RTK via its central Src homology (SH)-2 domain. On either side of the SH2 domain are SH3 domains, which bind to proline-rich regions of signaling proteins such as guanine exchange factor son-of-sevenless (SOS). The recruitment of SOS to the plasma membrane by Grb2 is required for the activation of Ras, which in turn sets in motion MAPK/ERK and PI3K/Akt signaling for gene expression and cell proliferation [129] (Figure 3A). Given its involvement in oncogenic signaling and correlation with poor patient prognosis [130,131,132], many anti-Grb2 therapeutic strategies are under development. Antagonists that bind to the Grb2′s SH2 domain, such as NHD2–15, have shown to inhibit the proliferation and metabolism of human leukemia cells in pre-clinical studies [133,134]. Although promising, to date only one Grb2 inhibitor is in clinical trial: BP1001, which utilizes ASO technology and liposomal delivery. 

### 4.1. BP1001 Pre-Clinical Studies

BP1001 consists of ASOs targeting the translation initiation site of Grb2 mRNA enclosed within a liposome. The 18-base ASO has a P-ethoxy backbone, providing it with nuclease resistance and high target affinity. Its neutral charge and hydrophobic nature facilitate its incorporation into liposomes, improving overall drug efficacy. The liposomes are formulated with neutral lipids, dioleoylphosphatidylcholine (DOPC) phospholipids and Tween 20 surfactant, to optimize membrane stability, fluidity, and cargo release (Figure 3B). Thus, BP1001 is uniquely different from other liposomal oligo formulations, which typically utilize positively charged lipids to bind to the negatively charged oligonucleotides [135,136]. The neutral charge of DOPC lipids reduces liposomes’ interactions with charged plasma proteins, thereby increasing blood circulation time and cellular uptake [137,138]. Additionally, DOPC is non-immunogenic and has a very low fatty acid chain melting temperature (below freezing point), making it highly desirable for drug delivery. DOPC has even been utilized to deliver siRNA to solid tumors; one such formulation is being investigated in a phase 1 study (ClinicalTrials.gov, number: NCT01591356). Previous pharmacokinetics and tissue distribution studies of such DOPC-liposomal P-ethoxy-ASO in mice displayed no adverse effects and primary accumulation in the liver and spleen, encouraging their use for leukemia and lymphoma therapies [85]. Indeed, mice bearing Bcr-Abl-positive 32D leukemia xenografts treated with 15 mg/kg BP1001 survived twice as long as mice treated with control liposomes (44.2 ± 8.5 versus 20.4 ± 7.9 days) [139].

In addition to hematological tumors, BP1001 provided similar results in solid tumors. When treated with BP1001, triple-negative breast cancer cells overexpressing EGFR displayed a reduction in Grb2 protein levels and proliferation [140]. In an orthotopic mouse model of ovarian cancer (OVCAR5 cells), bi-weekly injections of 15 mg/kg BP1001 resulted in a 70% reduction in tumor growth and 36% reduction in metastatic nodules. A BP1001 and paclitaxel combination treatment had enhanced therapeutic efficacy, with an 83% and 78% decrease in tumor growth and metastatic nodules, respectively [141]. Tumor growth in orthotopic models of uterine cancer was also decreased when treated with BP1001 alone (30% decrease) or in triple combination with paclitaxel and bevacizumab (77% decrease). The number of metastatic nodules also decreased by 73% following triple combination treatments. None of the drug treatments above showed signs of toxicity, as neither mouse weight nor gross morphology alterations were noticed compared to control mice, once again confirming BP1001 safety [141].

Although BP1001 is already in clinical trials for hematological malignancies, these equally promising pre-clinical results for solid tumors suggest that BP1001 may be a suitable candidate for ovarian and uterine cancer combination therapies.

### 4.2. BP1001 Clinical Trials

The phase 1 clinical trial of BP1001 (NCT01159028) consisted of a dose-escalating monotherapy to assess its pharmacokinetics, efficacy, and safety in adult patients with refractory/relapsed acute myeloid leukemia (AML), chronic myelogenous leukemia (CML), acute lymphoid leukemia, or myelodysplastic syndrome. Adopting a 3 + 3 dose-escalation strategy, the first cohort of patients started at a 5 mg/m^2^ dose of BP1001 administered intravenously twice weekly for 28 days. An absence of dose-limiting toxicities (DLTs) allowed patients to be sequentially enrolled in additional cohorts at 10 mg/m^2^ (Cohort 2), 20 mg/m^2^ (Cohort 3), 40 mg/m^2^ (Cohort 4), 60 mg/m^2^ (Cohort 5), and 90 mg/m^2^ (Cohort 6). Of the 21 patients assessable for DLTs, only one patient in cohort 1 displayed grade 4 mucositis and hand–foot syndrome while simultaneously taking hydroxyurea for highly proliferative CML in blast phase. No other patients experienced significant DLT, required BP1001 dose reductions or removal from the trial, or died due to drug toxicities, making the maximum tolerated dose (MTD) of BP1001 undetermined. These unique results may be attributed to the P-ethoxy oligonucleotide backbone, as ASOs with other chemical modifications (PS, GAPmer, LNA, ribose modifications) have been associated with coagulation, complement activation, and elevated levels of serum transaminases, indicative of liver damage [83,142,143]. One cycle of BP1001 monotherapy (eight doses) successfully reduced the number of peripheral blood blasts in 33% of patients and bone marrow blasts in 10% of patients by at least 50%. Twenty-two percent of patients benefited from monotherapy, exhibiting improved or stable disease, and received extended cycles of BP1001 treatment: Four patients completed two cycles and three completed five cycles [144]. 

The phase 1b trial involved the assessment of BP1001 treatments at 60 mg/m^2^ and 90 mg/m^2^ in combination with low-dose cytarabine, which was administered twice daily from day 10 to 19 of the BP1001 cycle. All seven AML patients enrolled tolerated combination treatments well, with no DLT detected. The decrease in bone marrow blasts was significantly higher (83%) in combination treatments compared to monotherapies, suggesting BP1001 to be efficacious in anti-leukemic combination therapies. The median overall survival of patients receiving combination therapy was approximately three times longer than those receiving BP1001 monotherapy (6.21 months vs 2.5 months) [144].

Pharmacokinetic analysis showed that the highest level of BP1001 in the blood and the extent of drug exposure over time was similar between the 60 mg/m^2^ and 90 mg/m^2^ doses. This indicates that BP1001 absorption is limited at doses higher than 60 mg/m^2^ and higher doses may be cleared more readily from the body. Grb2 levels in peripheral blood cells were monitored by flow cytometry at baseline, day 15, and at the end of treatments. Across all samples, 15 days post-treatment Grb2 levels decreased by a median of 54%, and by a median of 42% at the end of treatments [144].

These promising therapeutic and safety results have made BP1001 an appropriate anti-leukemic treatment option to pursue. A phase 2 clinical trial (NCT02781883) is currently recruiting newly diagnosed as well as refractory/relapsed AML participants to assess BP1001 and decitabine combination treatments as well as triple combinations of BP1001, decitabine, and venetoclax. BP1001′s efficacy against advanced/recurrent solid tumors will also be evaluated in a phase 1/1b clinical trial (NCT04196257). The MTD and potential toxicities will be determined in dose-escalating BP1001 monotherapies as well as paclitaxel combination treatments.

## 5. Future Perspectives

The use of oligonucleotides to manipulate protein production has become a prominent therapeutic strategy for the treatment of genetic disorders and cancer. The delivery of oligonucleotide drugs has required the development of chemically modified nucleic acids, bioconjugation to escort moieties, and the formulation of nanoparticle carriers. Although these technological advances have led to the clinical approval of several drugs, efficient and targeted delivery remains a major challenge. Liposomal nanoparticles address such a challenge. Liposomes are versatile and biocompatible, have low immunogenicity, show stability in the blood, and protect cargos from degradation. BP1001 is a neutral P-ethoxy-Grb2-ASO incorporated in neutral, un-tagged DOPC liposomes. With efficient anti-leukemic activity and a low toxicity profile in phase 1 clinical trials, this liposomal formulation is now being assessed in combination treatments (phase 2) and in a pending phase 1 study in solid tumors. Its use has also been extended to the delivery of other ASOs. Recently, BP1002, which targets anti-apoptotic Bcl-2, entered phase 1 clinical trials for the treatment of patients with advanced lymphoid malignancies (NCT04072458). Despite promising results that BP1001′s low toxicity is outbalanced by its efficacy, a twice-weekly intravenous injection schedule is not convenient for outpatients, indicating that liposomal formulations can yet be optimized for enhanced plasma stability. 

Anti-drug antibodies (ADAs) were reported in some clinical trials in patients following chronic treatment of antisense drugs [145]. ADAs against Mipomersen were detected in 4% of patients after 13 weeks of treatment and in 33% of patients after 50 weeks of treatment, thus demonstrating slow ADA development time [145]. The medical significance of ADAs is not known; it is possible that a high titer of ADAs could potentially reduce the efficacy of antisense drugs by lowering antisense concentration in plasma [145]. It is not clear whether ADAs will be detected in patients undergoing BP1001 treatment, as ADAs have not been reported in patients receiving morpholino drug treatments, suggesting that the appearance of ADAs may depend on antisense chemistry [145].

Another innovative technology currently under development is that of exosome-like nanoparticles. Similar to liposomes, these are purified or synthetic exosomes made of a lipid bilayer containing either entire cell-membrane patches or just selected membrane proteins for optimized delivery and targeting [99]. Although a promising alternative, with the advantage of effectively overcoming the blood-brain barrier, optimization of their formulations and stability are still underway.

Regardless of the delivery challenges, ASO therapeutics are paving the way in targeted and personalized medicine. Our growing understanding of disease genomics and identification of disease variants has allowed the design of ASOs to specifically correct gene mutations and control RNA expression, making them a therapeutic milestone in gene-specific therapies.

## Data Availability

Not applicable.
